# The dynamical perspective of soliton solutions, bifurcation, chaotic and sensitivity analysis to the (3+1)-dimensional Boussinesq model

**DOI:** 10.1038/s41598-024-59832-8

**Published:** 2024-04-22

**Authors:** Muhammad Nadeem, Asad Islam, Mehmet Şenol, Yahya Alsayaad

**Affiliations:** 1 School of Mathematics and Statistics, Qujing Normal University, Qujing, 655011 China; 2 Department of Mechanical and Aerospace Engineering, Air University, Islamabad, Pakistan; 3 Department of Mathematics, Nevşehir Hacı Bektaş Veli University, Nevşehir, Turkey; 4 Department of Physics, Hodeidah University, Al-Hudaydah, Yemen

**Keywords:** (3+1)-dimensional Boussinesq model, Unified Riccati equation expansion method, Exact soliton solutions, Bifurcation analysis, Chaotic structures, Sensitivity analysis, Quasi-periodic structures, Applied mathematics, Software

## Abstract

In this study, we examine multiple perspectives on soliton solutions to the (3+1)-dimensional Boussinesq model by applying the unified Riccati equation expansion (UREE) approach. The Boussinesq model examines wave propagation in shallow water, which is derived from the fluid dynamics of a dynamical system. The UREE approach allows us to derive a range of distinct solutions, such as single, periodic, dark, and rational wave solutions. Furthermore, we present the bifurcation, chaotic, and sensitivity analysis of the proposed model. We use planar dynamical system theory to analyze the structure and characteristics of the system’s phase portraits. The current study depends on a dynamic structure that has novel and unexplored results for this model. In addition, we display the behaviors of associated physical models in 3-dimensional, density, and 2-dimensional graphical structures. Our findings demonstrate that the UREE technique is a valuable mathematical tool in engineering and applied mathematics for studying wave propagation in nonlinear evolution equations.

## Introduction

In recent decades, the study of nonlinear evolution equations (NLEEs) have achieved great significance in various phenomena of science and engineering problems. The development of solutions to these NLEEs have emerged within the realm of nonlinear science, such as chemical physics, optical fiber, solid-state physics, and geochemistry^1–3^. Several studies have been reported on the solution of NLEEs in a technical and scientific domains. Numerous areas of mathematics and engineering are included; such as magnetism, hydrodynamics, thermal capacity, quantum dynamics, seismic waves, and the propagation of oceans^4–7^. Therefore, it is imperative to acquire analytical strategies for these NLEEs and show an extensive understanding of the qualitative features of such instances. Finding the analytical solution to these NLEEs have received a great attraction by various researchers.

Using symbolic tools, several suitable and efficient techniques for finding the appropriate solutions to various NLEEs have been demonstrated. There are several effective techniques, such as the Hirota bilinear technique (HBT)^8,9^, the modified Sardar sub-equation technique^10,11^, the hyperbolic-function technique^12^, the amended $\sinh$-Gordon expansion technique^13,14^, the Jacobi’s elliptic expansion technique^15,16^, the modified Fan-sub expansions technique^17,18^, the modified Kudryashov technique^19^, the Khater method^20^, the modified simple equation technique^21^, the unified Riccati equation expansion technique^22^ and so on^23,24^. The authors in^25^ studied the dynamical structure of soliton solutions, bifurcation analysis, and quasi-periodic solution to the (2+ 1)-dimensional Konopelchenko-Dubrovsky (KD) model. Kumar and Mann^26^ discussed the Schrödinger Boussinesq model to derive soliton solutions. Some potential scholars have recently solved several well-known NLEEs to find solutions using the HBT. For instance, the (3+1)-dimensional YTSF system^27^, the (3+1)-dimensional BKP-Boussinesq model^28^, the (2+1)-dimensional Burgers model^29^, the (3+1)-dimensional gKPB model^30^ and many more^31,32^.

In nonlinear sciences, the research of acoustic waves in shallow water is a widespread topic^33^. This type of acoustic wave is seen in streams, ocean coastlines, desert, and the water and can be explained by the Boussinesq model^34^, which is detailed below:

1 $$\begin{aligned} Q_{tt}-Q_{xx}-\alpha (Q^{2})_{xx}-\beta Q_{xxxx}=0. \end{aligned}$$

where *x*, *y*, *z* and *t* are spatial and temporal terms, *Q* is the dependent variable of the governing model. The Boussinesq model was initially established by Joseph Boussinesq in 1872 to explain how long, small-amplitude waves flow at a constant pace in a water channel with a constant depth. Additionally; the Boussinesq model is extensively applied in marine and coastal engineerings^35^. Because the Boussinesq model accurately models viscous flows involving various fluids with interfaces between them, the authors have considered it. Similar to viscous stresses, this model offers a fair distribution of turbulent stresses related to mean velocity gradients. It is also applied in road and geotechnical engineering for problems involving the distribution of vertical tension in soil medium. Because Boussinesq’s approximation ignores density fluctuations in inertial terms, it is frequently used in fluid dynamics with mild density gradients, which makes it appropriate for some applications.

The Boussinesq model is frequently used in coastal and ocean engineering to predict wave propagation in shallow coastlines and seas^36^. Now comprehend the dynamics of wave propagations on the ocean surfaces, our goal is to construct the exact solutions to a novel integrable (3+1)-dimensional Boussinesq model by implementing the unified Riccati equation expansion technique^37^. We take into consideration the innovative (3+1)-dimensional Boussinesq model^38^ to accomplish these goals.

2 $$\begin{aligned} Q_{tt}-Q_{xx}-\alpha (Q^{2})_{xx}-\beta Q_{xxxx}+\frac{\gamma ^{2}}{4}Q_{yy}+\gamma Q_{yt}+ \sigma Q_{xz}=0, \end{aligned}$$

where $\alpha ,~\beta ,~\gamma$ and $\sigma$ are constants and (*x*,  *y*,  *z*) and *t* shows the spatial and temporal parameters, respectively. By employing Hirota bilinear technique in 2019, Wazwaz and Kaur^39^ created several solution to Eq. (2) and then used the $exp(-\Phi (\zeta ))$-expansion technique to establish certain analytical solution. It is crucial to note that Eq. (2) can be transformed to the typical fourth-order Boussinesq model, which is provided by Eq. (1), if the value of $\sigma =0$ in Eq. (2), then transforms into a new integrable (2+1)-dimensional Boussinesq model. Thus, if we take into account $~\beta = 1,\alpha = 1,~\gamma = 1$ and $\sigma =1$, then Eq. (2), become

3 $$\begin{aligned} Q_{tt}-Q_{xx}-(Q^{2})_{xx}-Q_{xxxx}+\frac{1}{4}Q_{yy}+Q_{yt}+Q_{xz}=0, \end{aligned}$$

In this work, Eq. (3) can be transformed to (2+1)-dimensional Boussinesq model when $z=x$ and $z=y$. The lump solution to various dimensionally reduced equations have previously been discovered by some researchers^40–42^. In particular, Kaur and Wazwaz^28^ explored the lump solution to the reduced equations of (3+1)-dimensional generalized Boussinesq model. Singh et al.^41^ found results for the novel (2+1)-dimensional Boussinesq model, including both brilliant and dark rogue waves. Huang et al.^42^ discovered the lump waveform and kink soliton solution for the generalized (3+1)-dimensional KP model. The physical phenomena can be enhanced by using the visual representation of the solutions to the Boussinesq model, which highlights the physical characteristics of the model. Understanding the solutions to real-world problems, such as ocean waves, aids us in accurately comprehending them. It is important to emphasize that all results show novel properties emerging in shallow water waves.

Our literature analysis indicates that the UREE technique has not been previously employed to investigate the transmission of optical solitons in optical fibers. In our analysis, we have also considered bifurcation analysis, physical features, and the application of findings to better understand the dynamic processes of the model. Through the extension of the Riccati equation, a methodical technique is employed in the process. An approximate version of the solutions known as the general ansatz is used that subsequently entered into the formula. The differential equation is converted into a nonlinear algebraic equation using algebraic operations. After that, new soliton solutions can be obtained by solving these nonlinear algebraic equations. Our objective for this study is to evaluate the soliton solutions of the (3+1)-dimensional Boussinesq model using a novel technique. These are the primary sections of this research. The unified Riccati equation expansion (UREE) method is described in Section “Description of the UREE technique”. The derivation of the soliton solutions has been stated in Section “Implementation”. In Section “Bifurcation analysis”, the phase portraits of bifurcations are utilized to illustrate the qualitative behavior of the analyzed model. In Section “Results and discussions”, we present some discussions on the obtained results. Finally, Section “Conclusion” covers the conclusion part of this study.

## Description of the UREE technique

The generic form of NLEEs is

4 $$\begin{aligned} R(f,f_{x},f_{y},f_{xx},\ldots )=0. \end{aligned}$$

***Step-i:*** Utilize the transformations of wave, such that

5 $$\begin{aligned} Q=q(\xi ){,}~~~~~~~~~\text {and}~~~~~~~~~\xi =x+y+nz-mt, \end{aligned}$$

be used in Eq. (4), then we can obtain the nonlinear ordinary differential equation (NLODE) as,

6 $$\begin{aligned} {\mathcal {U}}(f,f^{\prime },f^{\prime \prime },\ldots )=0. \end{aligned}$$

***Step-ii:*** According to Eq. (6), the general solution is

7 $$\begin{aligned} q(\xi )=s_{0}+\sum _{d=1}^{M }s_{d}(\phi (\xi )^d), \end{aligned}$$

as $s_{0},~s_{d}$ are necessary to compute and $s_{M}\ne 0$ and $\phi (\xi )$ satisfy the ODE as below

8 $$\begin{aligned} \phi '(\xi )= f_0 + f_1 \phi (\xi )+ f_2 \phi (\xi )^2. \end{aligned}$$

***Step-iii:*** We acquired a positive number *M*, by applying the balancing principle as in Eq. (7).

***Step-iv:*** By resolving Eqs. (7) and (8) into Eq. (6), we acquire a system of equations and gather all the terms that equal zero and have the same power of $(\phi (\xi )^d)$. $s_{0},s_{1},~f_{0},~f_{1}$ and *n* are the values that are obtained for carrying out a symbolic solution of the provided model. Below are the solutions to Eq. (8).

**Cluster-(a): If** $${\mathcal {L}}>0$$

9 $$\begin{aligned} \phi _1(\xi )= & {} -\frac{f_1}{2 f_2}-\frac{\sqrt{{\mathcal {L}}} \tanh \left( \frac{\xi \sqrt{{\mathcal {L}}}}{2}\right) }{2 f_2}, \end{aligned}$$

10 $$\begin{aligned} \phi _2(\xi )= & {} -\frac{f_1}{2 f_2}-\frac{\sqrt{{\mathcal {L}}} \coth \left( \frac{\xi \sqrt{{\mathcal {L}}}}{2}\right) }{2 f_2}. \end{aligned}$$

**Cluster-(b): If** $${\mathcal {L}}<0$$

11 $$\begin{aligned} \phi _3(\xi )= & {} -\frac{f_1}{2 f_2}-\frac{\sqrt{-{\mathcal {L}}} \tan \left( \frac{\xi \sqrt{-{\mathcal {L}}}}{2}\right) }{2 f_2}, \end{aligned}$$

12 $$\begin{aligned} \phi _4(\xi )= & {} -\frac{f_1}{2 f_2}-\frac{\sqrt{-{\mathcal {L}}} \cot \left( \frac{\xi \sqrt{-{\mathcal {L}}}}{2}\right) }{2 f_2}. \end{aligned}$$

**Cluster-(c): If** $${\mathcal {L}}=0$$

13 $$\begin{aligned} \phi _5(\xi )=-\frac{1}{e_1+f_2 \xi }-\frac{f_1}{2 f_2}. \end{aligned}$$

***Step-v:*** The parametric values and the solution of Eq. (8) reinserted into Eq. (7) allow us to find the precise solutions to Eq. (4).

## Implementation

The main focus of this part is the application of our suggested technique to verify its performance, efficacy, and dependability. It will offer us a selection of solutions for the Boussinesq model, which is integrable in the (3+1)-dimensional space. The following transformation is provided in Eq. (5). Next, the transformation provided in Eq. (5) is applied to transform Eq. (3) into NLODE. Thus

14 $$\begin{aligned} \begin{aligned} m^2 q''(\xi )-q^{(4)}(\xi )-m q''(\xi )-\frac{3}{4} q''(\xi )+n q''(\xi )-2 q(\xi ) q''(\xi )-2 q'(\xi )^2=0. \end{aligned} \end{aligned}$$

Integrating twice Eq. (14), to get the required second-order ordinary differential equation

15 $$\begin{aligned} \begin{aligned} \frac{1}{4} \left( 4 m^2-4 m+4 n-3\right) q(\xi )-q''(\xi )-q(\xi )^2=0. \end{aligned} \end{aligned}$$

By using the balancing principle from Eq. (15), we can get $M=2$. Let $M=2$, the general solution of Eq. (7) becomes

16 $$\begin{aligned} q(\xi )= s_0 + s_1 \phi (\xi )+ s_2 \phi (\xi )^2. \end{aligned}$$

Now, equating the coefficients of same power of $(\phi (\xi ))^{d}$, where $d=0,1,2,3,\ldots$. The Eq. (16) is incorporated into Eq. (15) with Eq. (8) and thus we can obtain a system of algebraic equations such as

17 $$\begin{aligned} \begin{aligned} -f_0 f_1 s_1-2 f_0^2 s_2+m^2 s_0-m s_0+n s_0-s_0^2-\frac{3 s_0}{4}=0,~~~~~~~~~~~~~~~ \\ +\left( -f_1^2 s_1-2 f_0 f_2 s_1-6 f_0 f_1 s_2+m^2 s_1-m s_1+n s_1-2 s_0 s_1-\frac{3 s_1}{4}\right) =0, \\+\left( -3 f_1 f_2 s_1-4 f_1^2 s_2-8 f_0 f_2 s_2+m^2 s_2-m s_2+n s_2-s_1^2-2 s_0 s_2-\frac{3 s_2}{4}\right) =0 \\+\left( -2 f_2^2 s_1-10 f_1 f_2 s_2-2 s_1 s_2\right) =0,~~~~~~~~~~~~~~~~~~~~~~~~~ \\+\left( -6 f_2^2 s_2-s_2^2\right) =0.~~~~~~~~~~~~~~~~~~~~~~~~~~~~~~ \end{aligned} \end{aligned}$$

Using symbolic computing, we solve the above system of equations and hence we can obtain a set of solutions as follows

***Family-1:***

18 $$\begin{aligned} \begin{aligned} \Bigg \{s_0\rightarrow -i \sqrt{\frac{2}{3}} f_0 \sqrt{s_2},~~~s_1\rightarrow 0,~~~f_1\rightarrow 0,~~~f_2\rightarrow \frac{i \sqrt{s_2}}{\sqrt{6}},~~~n\rightarrow 2 i \sqrt{\frac{2}{3}} f_0 \sqrt{s_2}-m^2+m+\frac{3}{4}\Bigg \}{.} \end{aligned} \end{aligned}$$

According to the results, Family-1 is satisfied by the following solutions.

- When ${\mathcal {L}}>0$, we get
  19 $$\begin{aligned}{} & {} \begin{aligned} Q_{1,1}(x,t)=\frac{i \sqrt{\frac{3}{2}} \sqrt{{\mathcal {L}}} \tanh \left( \frac{1}{2} \sqrt{{\mathcal {L}}} \left( z \left( 2 i \sqrt{\frac{2}{3}} f_0 \sqrt{s_2}-m^2+m+\frac{3}{4}\right) -m t+x+y\right) \right) }{\sqrt{s_2}}, \end{aligned} \end{aligned}$$
  20 $$\begin{aligned}{} & {} \begin{aligned} Q_{1,2}(x,t)=\frac{i \sqrt{\frac{3}{2}} \sqrt{{\mathcal {L}}} \coth \left( \frac{1}{2} \sqrt{{\mathcal {L}}} \left( z \left( 2 i \sqrt{\frac{2}{3}} f_0 \sqrt{s_2}-m^2+m+\frac{3}{4}\right) -m t+x+y\right) \right) }{\sqrt{s_2}}. \end{aligned} \end{aligned}$$
- When ${\mathcal {L}}<0$, we get
  21 $$\begin{aligned}{} & {} \begin{aligned} Q_{1,3}(x,t)=\frac{i \sqrt{\frac{3}{2}} \sqrt{-{\mathcal {L}}} \tan \left( \frac{1}{2} \sqrt{-{\mathcal {L}}} \left( z \left( 2 i \sqrt{\frac{2}{3}} f_0 \sqrt{s_2}-m^2+m+\frac{3}{4}\right) -m t+x+y\right) \right) }{\sqrt{s_2}}, \end{aligned} \end{aligned}$$
  22 $$\begin{aligned}{} & {} \begin{aligned} Q_{1,4}(x,t)=\frac{i \sqrt{\frac{3}{2}} \sqrt{-{\mathcal {L}}} \cot \left( \frac{1}{2} \sqrt{-{\mathcal {L}}} \left( z \left( 2 i \sqrt{\frac{2}{3}} f_0 \sqrt{s_2}-m^2+m+\frac{3}{4}\right) -m t+x+y\right) \right) }{\sqrt{s_2}}. \end{aligned} \end{aligned}$$
- When ${\mathcal {L}}=0$, we get
  23 $$\begin{aligned} \begin{aligned} Q_{1,5}(x,t)=-\frac{1}{e_1+\frac{i \sqrt{s_2} \left( z \left( 2 i \sqrt{\frac{2}{3}} f_0 \sqrt{s_2}-m^2+m+\frac{3}{4}\right) -m t+x+y\right) }{\sqrt{6}}}. \end{aligned} \end{aligned}$$

***Family-2:***

24 $$\begin{aligned} \begin{aligned} \Bigg \{s_0\rightarrow \frac{s_1^2+2 i \sqrt{6} f_0 s_2^{3/2}}{6 s_2},~~~f_1\rightarrow -\frac{i s_1}{\sqrt{6} \sqrt{s_2}},~~~f_2\rightarrow -\frac{i \sqrt{s_2}}{\sqrt{6}},~~~n\rightarrow \frac{s_1^2-4 i \sqrt{6} f_0 s_2^{3/2}}{6 s_2}-m^2+m+\frac{3}{4}\Bigg \}{.} \end{aligned} \end{aligned}$$

According to the results, Family-2 is satisfied by the following solutions.

- When ${\mathcal {L}}>0$, we get
  25 $$\begin{aligned}{} & {} \begin{aligned} Q_{2,1}(x,t)=-\frac{s_1}{2 s_2}-\frac{i \sqrt{\frac{3}{2}} \sqrt{{\mathcal {L}}} \tanh \left( \frac{1}{2} \sqrt{{\mathcal {L}}} \left( z \left( \frac{s_1^2-4 i \sqrt{6} f_0 s_2^{3/2}}{6 s_2}-m^2+m+\frac{3}{4}\right) -m t+x+y\right) \right) }{\sqrt{s_2}}, \end{aligned} \end{aligned}$$
  26 $$\begin{aligned}{} & {} \begin{aligned} Q_{2,2}(x,t)=-\frac{s_1}{2 s_2}-\frac{i \sqrt{\frac{3}{2}} \sqrt{{\mathcal {L}}} \coth \left( \frac{1}{2} \sqrt{{\mathcal {L}}} \left( z \left( \frac{s_1^2-4 i \sqrt{6} f_0 s_2^{3/2}}{6 s_2}-m^2+m+\frac{3}{4}\right) -m t+x+y\right) \right) }{\sqrt{s_2}}. \end{aligned} \end{aligned}$$
- When ${\mathcal {L}}<0$, we get
  27 $$\begin{aligned}{} & {} \begin{aligned} Q_{2,3}(x,t)=-\frac{s_1}{2 s_2}-\frac{i \sqrt{\frac{3}{2}} \sqrt{-{\mathcal {L}}} \tan \left( \frac{1}{2} \sqrt{-{\mathcal {L}}} \left( z \left( \frac{s_1^2-4 i \sqrt{6} f_0 s_2^{3/2}}{6 s_2}-m^2+m+\frac{3}{4}\right) -m t+x+y\right) \right) }{\sqrt{s_2}}, \end{aligned} \end{aligned}$$
  28 $$\begin{aligned}{} & {} \begin{aligned} Q_{2,4}(x,t)=-\frac{s_1}{2 s_2}-\frac{i \sqrt{\frac{3}{2}} \sqrt{-{\mathcal {L}}} \cot \left( \frac{1}{2} \sqrt{-{\mathcal {L}}} \left( z \left( \frac{s_1^2-4 i \sqrt{6} f_0 s_2^{3/2}}{6 s_2}-m^2+m+\frac{3}{4}\right) -m t+x+y\right) \right) }{\sqrt{s_2}}. \end{aligned} \end{aligned}$$
- When ${\mathcal {L}}=0$, we get
  29 $$\begin{aligned} \begin{aligned} Q_{2,5}(x,t)=-\frac{s_1}{2 s_2}-\frac{1}{e_1-\frac{i \sqrt{s_2} \left( z \left( \frac{s_1^2-4 i \sqrt{6} f_0 s_2^{3/2}}{6 s_2}-m^2+m+\frac{3}{4}\right) -m t+x+y\right) }{\sqrt{6}}}. \end{aligned} \end{aligned}$$

***Family-3:***

30 $$\begin{aligned} \begin{aligned} \Bigg \{s_0\rightarrow -i \sqrt{6} f_0 \sqrt{s_2},~~~f_1\rightarrow \frac{i s_1}{\sqrt{6} \sqrt{s_2}},~~~f_2\rightarrow \frac{i \sqrt{s_2}}{\sqrt{6}},~~~n\rightarrow -\frac{s_1^2+4 i \sqrt{6} f_0 s_2^{3/2}}{6 s_2}-m^2+m+\frac{3}{4}\Bigg \}{.} \end{aligned} \end{aligned}$$

According to the results, Family-3 is satisfied by the following solutions.

- When ${\mathcal {L}}>0$, we get
  31 $$\begin{aligned}{} & {} \begin{aligned} Q_{3,1}(x,t)=-\frac{s_1}{2 s_2}+\frac{i \sqrt{\frac{3}{2}} \sqrt{{\mathcal {L}}} \tanh \left( \frac{1}{2} \sqrt{{\mathcal {L}}} \left( z \left( -\frac{s_1^2+4 i \sqrt{6} f_0 s_2^{3/2}}{6 s_2}-m^2+m+\frac{3}{4}\right) -m t+x+y\right) \right) }{\sqrt{s_2}}, \end{aligned} \end{aligned}$$
  32 $$\begin{aligned}{} & {} \begin{aligned} Q_{3,2}(x,t)=-\frac{s_1}{2 s_2}+\frac{i \sqrt{\frac{3}{2}} \sqrt{{\mathcal {L}}} \coth \left( \frac{1}{2} \sqrt{{\mathcal {L}}} \left( z \left( -\frac{s_1^2+4 i \sqrt{6} f_0 s_2^{3/2}}{6 s_2}-m^2+m+\frac{3}{4}\right) -m t+x+y\right) \right) }{\sqrt{s_2}}. \end{aligned} \end{aligned}$$
- When ${\mathcal {L}}<0$, we get
  33 $$\begin{aligned}{} & {} \begin{aligned} Q_{3,3}(x,t)=-\frac{s_1}{2 s_2}+\frac{i \sqrt{\frac{3}{2}} \sqrt{-{\mathcal {L}}} \tan \left( \frac{1}{2} \sqrt{-{\mathcal {L}}} \left( z \left( -\frac{s_1^2+4 i \sqrt{6} f_0 s_2^{3/2}}{6 s_2}-m^2+m+\frac{3}{4}\right) -m t+x+y\right) \right) }{\sqrt{s_2}}, \end{aligned} \end{aligned}$$
  34 $$\begin{aligned}{} & {} \begin{aligned} Q_{3,4}(x,t)=-\frac{s_1}{2 s_2}+\frac{i \sqrt{\frac{3}{2}} \sqrt{-{\mathcal {L}}} \cot \left( \frac{1}{2} \sqrt{-{\mathcal {L}}} \left( z \left( -\frac{s_1^2+4 i \sqrt{6} f_0 s_2^{3/2}}{6 s_2}-m^2+m+\frac{3}{4}\right) -m t+x+y\right) \right) }{\sqrt{s_2}}. \end{aligned} \end{aligned}$$
- When ${\mathcal {L}}=0$, we get
  35 $$\begin{aligned} \begin{aligned} Q_{3,5}(x,t)=-\frac{s_1}{2 s_2}-\frac{1}{e_1+\frac{i \sqrt{s_2} \left( z \left( -\frac{s_1^2+4 i \sqrt{6} f_0 s_2^{3/2}}{6 s_2}-m^2+m+\frac{3}{4}\right) -m t+x+y\right) }{\sqrt{6}}}. \end{aligned} \end{aligned}$$

**Figure 1:**
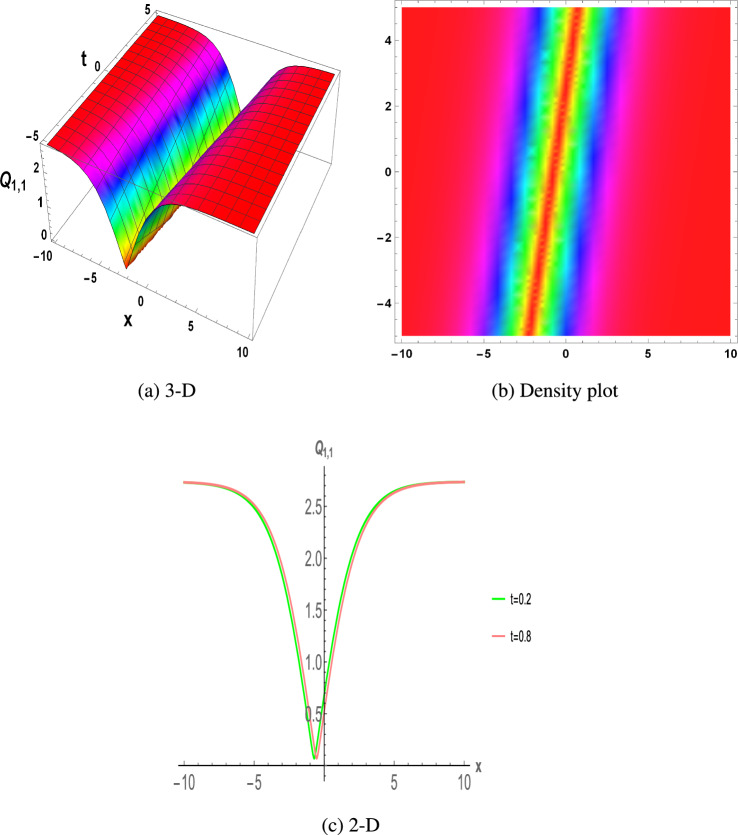
The parametric values ${\mathcal {L}} =1.25,~f_{0}=3.2,~m =1.23,~y=1.2,~z=1.43$ and $s_{2}=1.21$ depict the physical structure of dark solution of the $Q_{1,1}(x,t)$ in Eq. (19).

**Figure 2:**
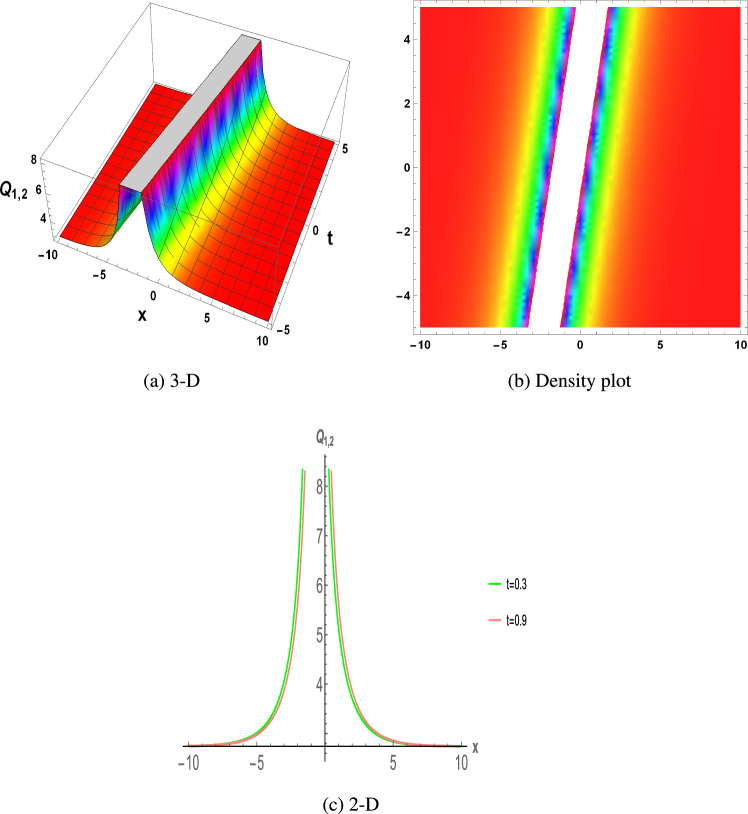
The parametric values ${\mathcal {L}} =1.5,~f_{0}=3.2,~m =2.03,~y=1.32,~z=1.23$ and $s_{2}=1.23$ depict the physical structure of singular solution of the $Q_{1,2}(x,t)$ in Eq. (20).

**Figure 3:**
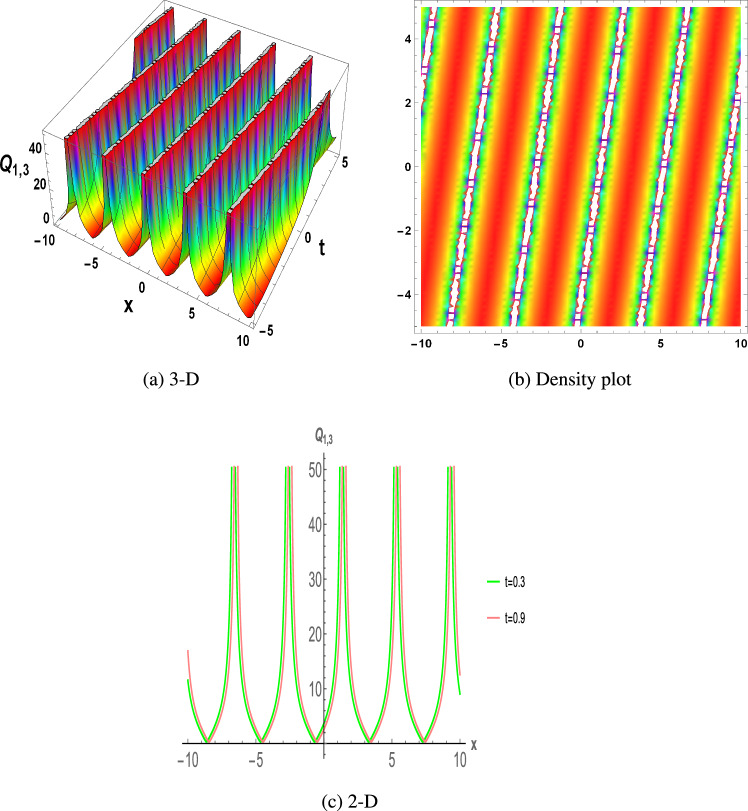
The parametric values ${\mathcal {L}} =-0.35,~f_{0}=3.2,~m =1.03,~y=1.2,~z=1.34$ and $s_{2}=1.31$ depict the physical structure of periodic solution of the $Q_{1,3}(x,t)$ in Eq. (21).

**Figure 4:**
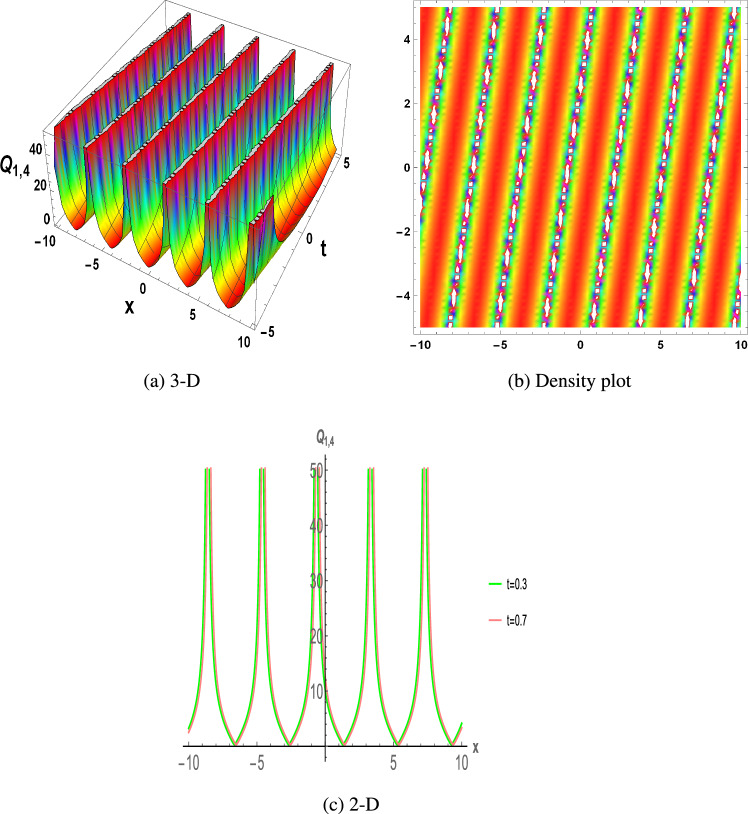
The parametric values ${\mathcal {L}} =-0.25,~f_{0}=2.2,~m =1.3,~y=1.21,~z=1.3$ and $s_{2}=0.34$ depict the physical structure of periodic solution of the $Q_{1,4}(x,t)$ in Eq. (22).

**Figure 5:**
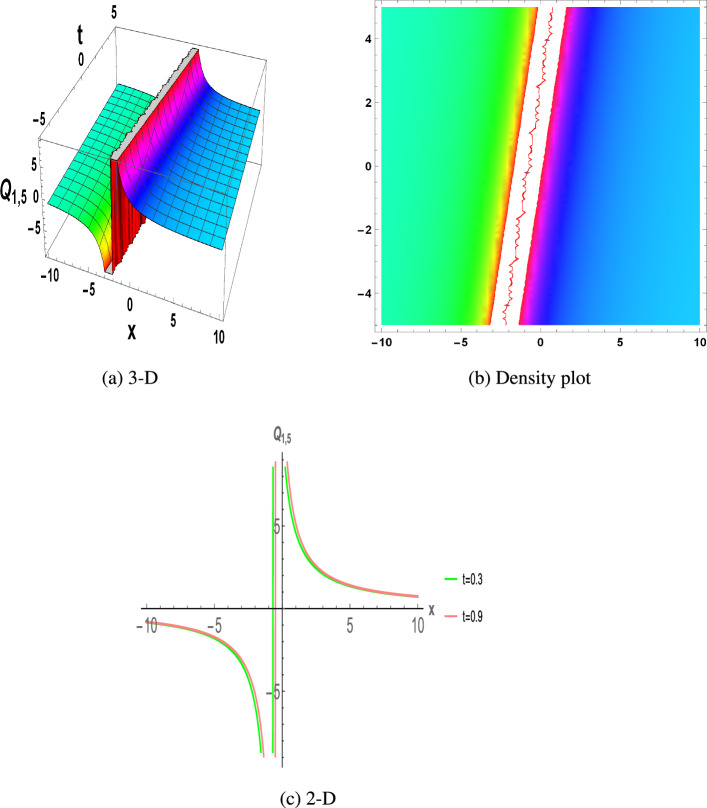
The parametric values ${\mathcal {L}} =0,~f_{0}=0.32,~m =1.3,~y=0.3,~z=1.3$ and $s_{2}=1.1$ depict the physical structure of rational solution of the $Q_{1,5}(x,t)$ in Eq. (23).

## Bifurcation analysis

In this section, we investigate Eq. (3) using bifurcation theory and phase portrait analysis^35^. The system described by Eq. (3) has its own dynamic influence, allowing us to see how changes in parameters affect the quality of the system. A framework for examining the bifurcations that occur within a family of systems is provided by bifurcation theory, which enables us to pinpoint the typical bifurcation patterns. By using the Galilean transformation, we may express the planar dynamical system for Eq. (15) as follows,

36 $$\begin{aligned}{} & {} \frac{dq}{d\xi }=p,~~ \nonumber \\{} & {} \frac{d^{2}q}{d\xi ^{2}}=p'{.} \end{aligned}$$

When applying the aforementioned transformation to Eq. (15), we obtain

$$\begin{aligned}{} & {} \frac{1}{4}(-3-4m+4m^{2}+4n)q-q^{2}-p'=0, \\{} & {} p'=\frac{1}{4}(-3-4m+4m^{2}+4n)q-q^{2}. \end{aligned}$$

37 $$\begin{aligned} \frac{dq}{d\xi }=p,~~~~~~~~~~~~~~~~~~~~~~~~~ \nonumber \\ \frac{d^{2}q}{d\xi ^{2}}=\frac{1}{4}(-3-4m+4m^{2}+4n)q-q^{2}. \end{aligned}$$

Assuming that *q* and *p* are the functions:

38 $$\begin{aligned} q'={\mathcal {H}}(q,p), \nonumber \\p'={\mathcal {G}}(q,p). \end{aligned}$$

As $p'=Cq-Dq^{2}$, where $C=\frac{1}{4}(-3-4m+4m^{2}+4n)$ and $D=1$. So that $q'=p$ and $q''=Cq-Dq^{2}$. That is

39 $$\begin{aligned}{} & {} {\mathcal {J}}(Q,P)=\begin{vmatrix} \frac{\partial {\mathcal {H}}}{\partial q}&\frac{\partial {\mathcal {H}}}{\partial p}\\ \frac{\partial {\mathcal {G}}}{\partial q}&\frac{\partial {\mathcal {G}}}{\partial p} \end{vmatrix}, \end{aligned}$$

40 $$\begin{aligned}{} & {} \begin{aligned} {\mathcal {J}}(Q,P)= \begin{vmatrix} 0&1\\ C-2Dq&0 \end{vmatrix} =-C+2Dq. \end{aligned} \end{aligned}$$

There are two equilibrium points for the equation $p'=Cq-Dq^{2}$: ${\mathcal {J}}_{1}(Q,P) = (0, 0)$ and ${\mathcal {J}}_{2}(Q,P) = (Q, 0)$. Therefore, (*Q*, 0) can be classified as a saddle point when ${\mathcal {J}}(Q, P)< 0$, a center when ${\mathcal {J}}(Q, P) > 0$ and a cuspidal point when ${\mathcal {J}}(Q, P) = 0$. Similar to this, if ${\mathcal {J}}(Q, P)<0$, ${\mathcal {J}}(Q, P)> 0$ and ${\mathcal {J}}(Q, P) = 0$, then (0, *p*) is a saddle point, a center and a cuspidal point respectively. It is significant to remember that *Q* and *P* can take real values depending on the specific choices made for the parameters. We experience many circumstances for various parameter choices, each of which will be discussed in detail.

**Figure 6:**
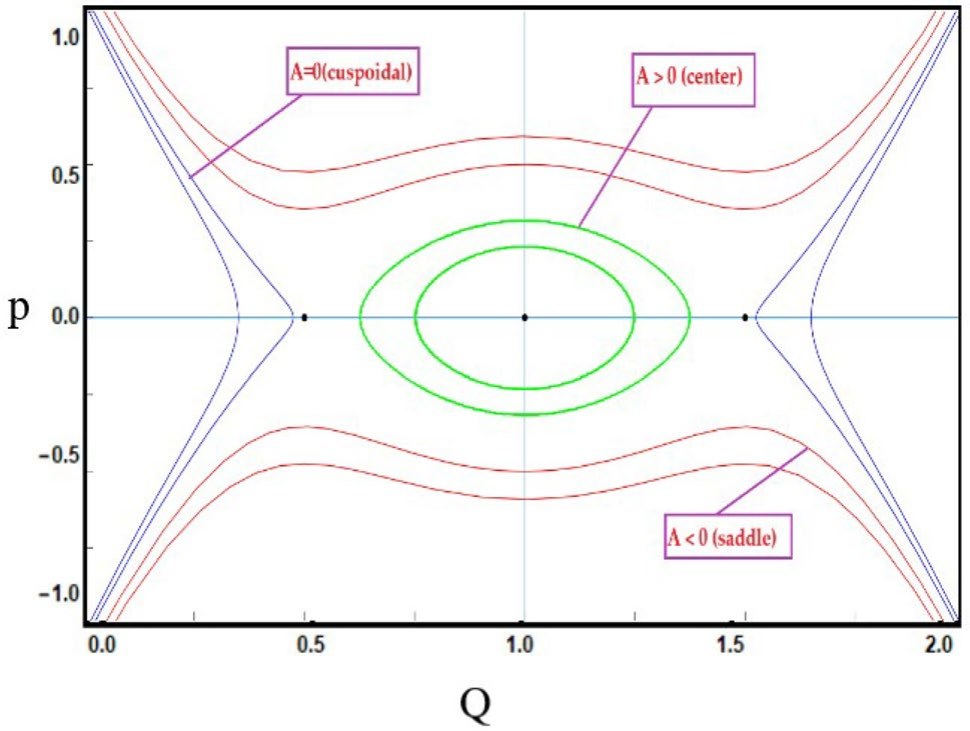
*Case-*1: Analysis of the phase depiction for $C> 0 ~ \&  ~D>0$.

***Case-1:*** For first equilibrium point ${\mathcal {J}}_{1}(0,0)$. If $C>0$ and $D>0$, then ${\mathcal {J}}_{1}(Q,P)$ get center point when $A={\mathcal {J}}(Q,P)>0$, ${\mathcal {J}}_{1}(Q,P)$ get saddle point when $A={\mathcal {J}}(Q,P)<0$ and ${\mathcal {J}}_{1}(Q,P)$ get cuspidal point when $A={\mathcal {J}}(Q,P)=0$. Similarly, for second equilibrium point ${\mathcal {J}}_{2}(\frac{C}{D},0)$, ${\mathcal {J}}_{2}(Q,P)$ get center point when ${\mathcal {J}}(Q,P)>0$, ${\mathcal {J}}_{2}(Q,P)$ get saddle point when ${\mathcal {J}}(Q,P)<0$ and ${\mathcal {J}}_{3}(Q,P)$ get cuspidal point when ${\mathcal {J}}(Q,P)=0$ as depict in Fig. 6.

**Figure 7:**
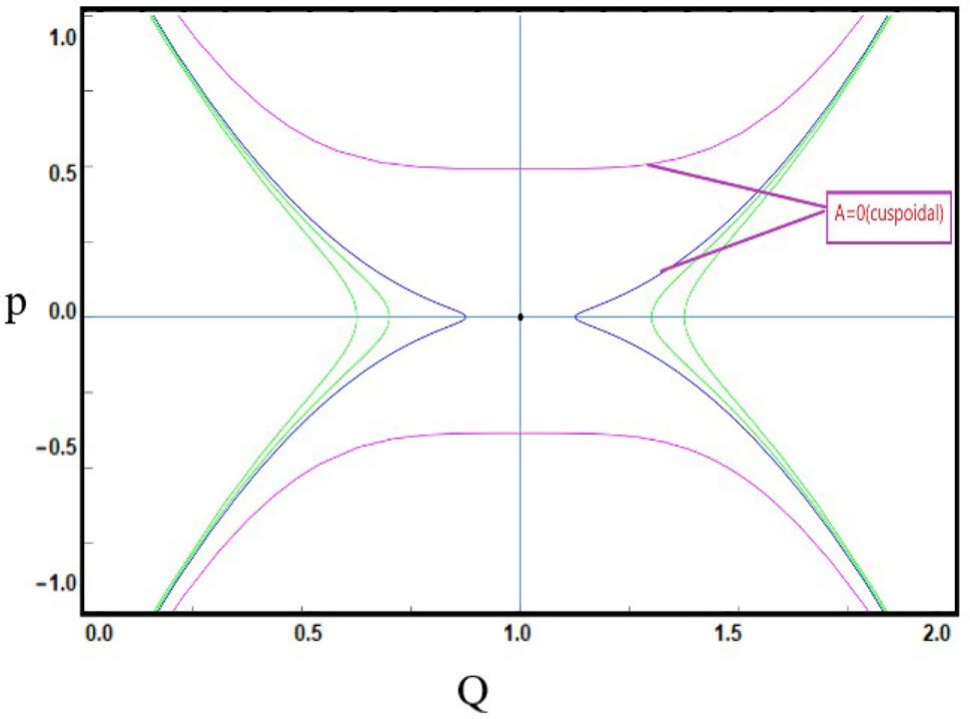
*Case-*2: Analysis of the phase depiction for $C=0~ \& ~D<0$.

***Case-2:*** If $C=0$ and $D<0$, then only one equilibrium point exists that is ${\mathcal {J}}_{1}(0,0)$. $A={\mathcal {J}}_{1}(Q,P)$ get cuspidal point when ${\mathcal {J}}(Q,P)=0$ as depict in Fig. 7.

**Figure 8:**
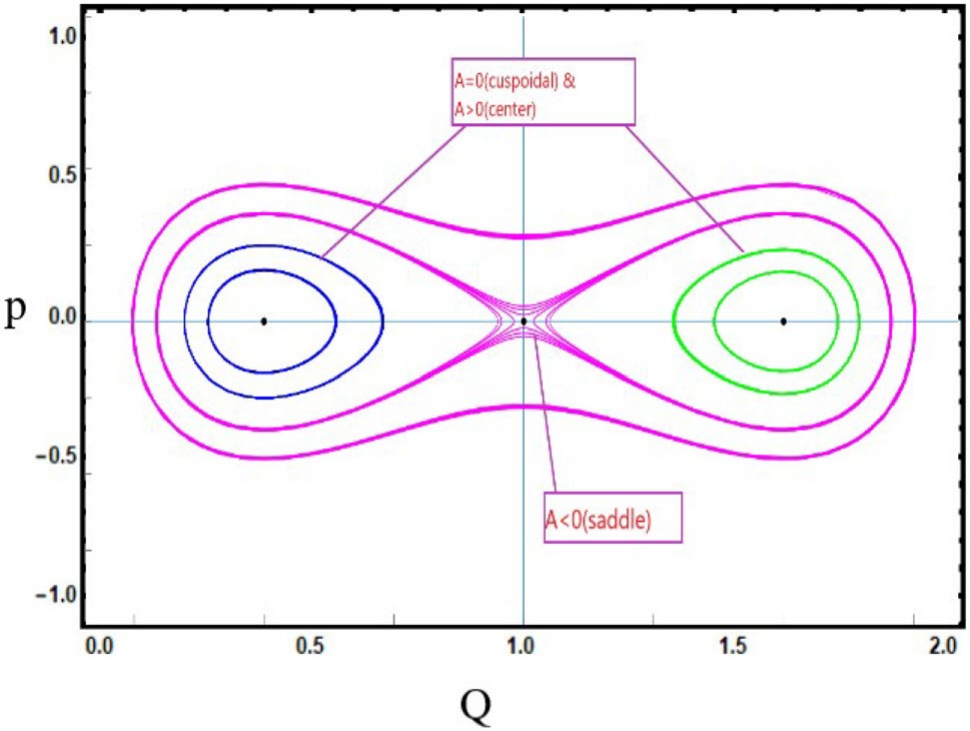
*Case-*3: Analysis of the phase depiction for $C<0~ \& ~D<0$.

***Case-3:*** If $C<0~,~D<0$, for first equilibrium point ${\mathcal {J}}_{1}(Q,P)$, then ${\mathcal {J}}_{1}(Q,P)$ get center point when $A={\mathcal {J}}(Q,P)>0$, ${\mathcal {J}}_{1}(Q,P)$ get saddle point when $A={\mathcal {J}}(Q,P)<0$ and ${\mathcal {J}}_{3}(Q,P)$ get cuspidal point when $A={\mathcal {J}}(Q,P)=0$. Similarly, for second equilibrium point ${\mathcal {J}}_{2}(\frac{C}{D},0)$, that is ${\mathcal {J}}_{2}(Q,P)$ get center point when $A={\mathcal {J}}(Q,P)>0$, ${\mathcal {J}}_{2}(Q,P)$ get saddle point when $A={\mathcal {J}}(Q,P)<0$ and ${\mathcal {J}}_{3}(Q,P)$ get cuspidal point when $A={\mathcal {J}}(Q,P)=0$ as depict in Fig. 8.

**Figure 9:**
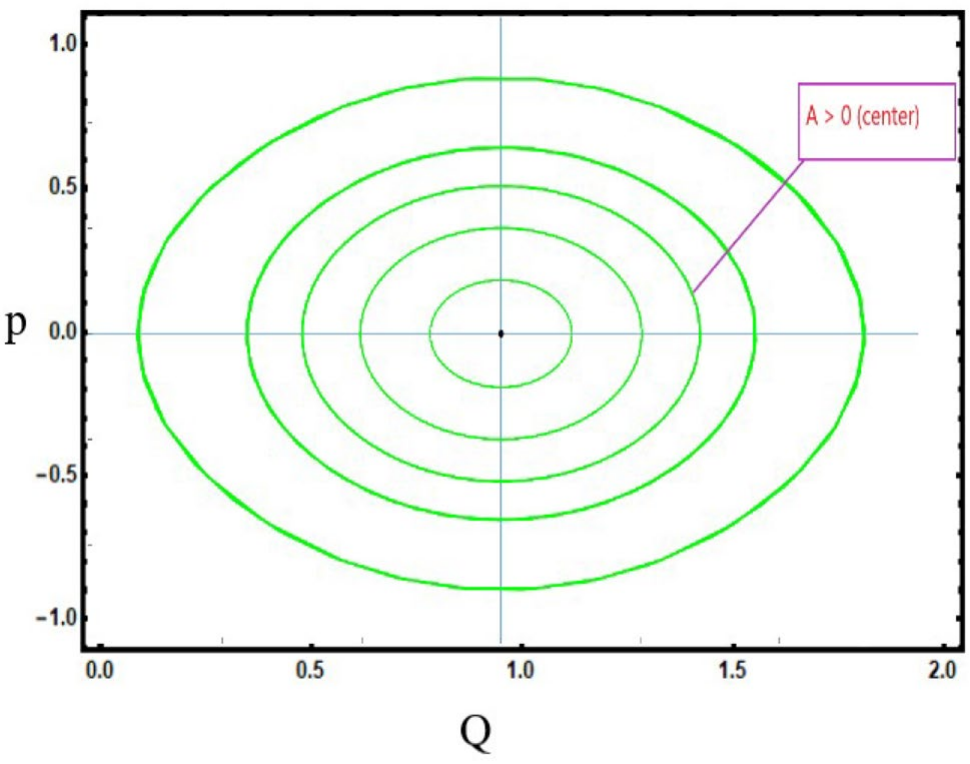
*Case-*4: Analysis of the phase depiction for $C>0~ \& ~D=0$.

***Case-4:*** For $C>0$ and $D<0$, If $C>0$ and $D<0$, then for only one equilibrium point exist that is ${\mathcal {J}}_{1}(0,0)$. $A={\mathcal {J}}_{1}(Q,P)$ is a center point if $A={\mathcal {J}}(Q,P)>0$ as shown in Fig. 9.

**Figure 10:**
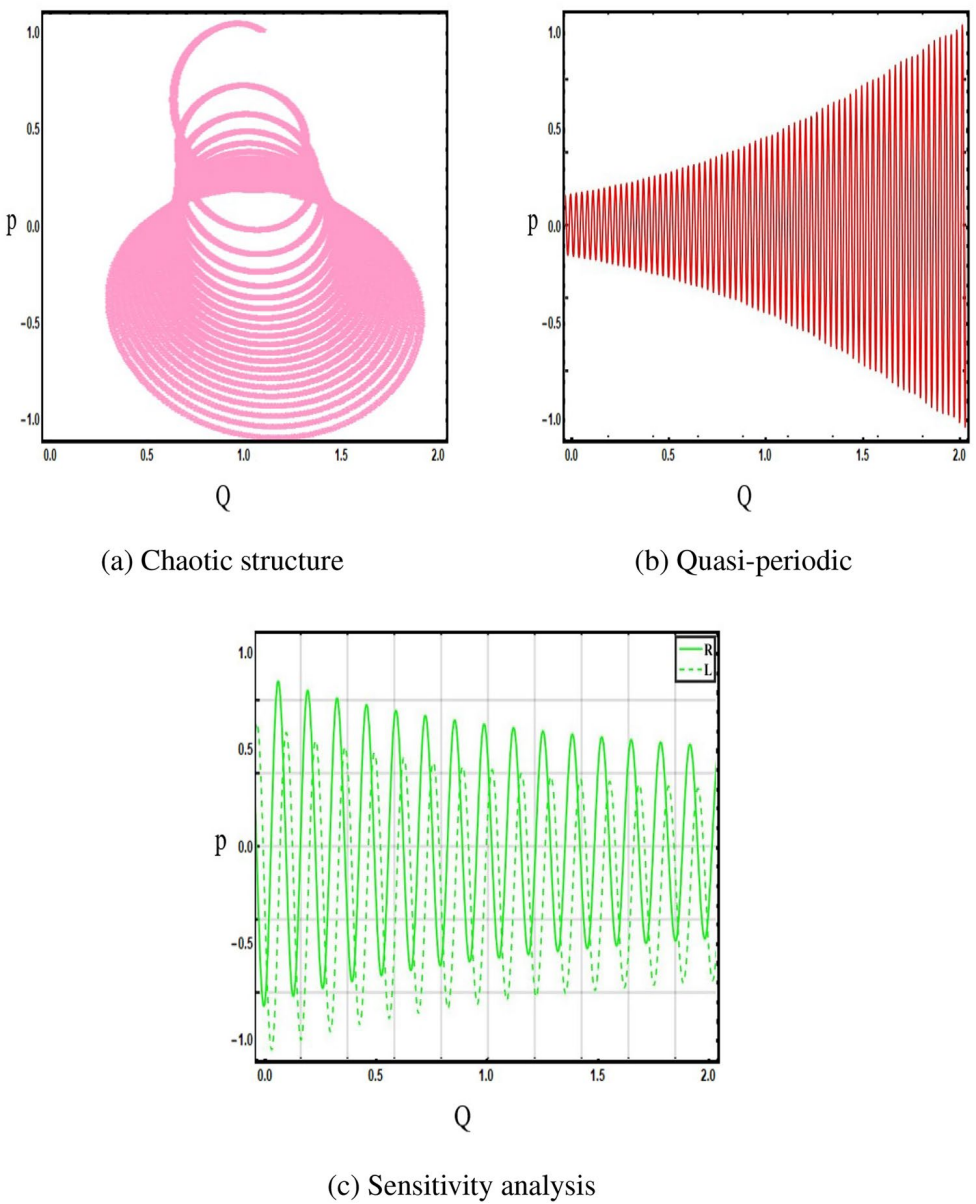
The illustration of Eq. (41) under Parametric values are $m=0.3, ~\Psi =0.4,~q=0.1,~\xi =1.2,$ and initial condition is $(Q,~P)=(0.1,~1.2).$.

Now add the perturbation term in Eq. (37), to discuss the chaotic, quasi-periodic and sensitivity analysis structures by using different values of frequency with initial conditions. Now, the chaotic equation below

41 $$\begin{aligned}{} & {} \frac{dq}{d\xi }=p, \nonumber \\{} & {} \frac{d^{2}q}{d\xi ^{2}}=\frac{1}{4}(-3-4m+4m^{2}+4n)q-q^{2}+ \Psi \sin (\Gamma \xi ), \end{aligned}$$

where $\Psi$ is frequency, $\Gamma$ is amplitude and $\xi$ is independent variable. The illustration of quasi-periodic, chaotic and sensitivity analysis as shown in Fig. 10 under suitable parametric values.

## Results and discussions

This section discuss on recent findings in conjunction with a comparison of previous study^38^ where the authors investigated the integrable (3+1)-dimensional Boussinesq model to obtain lump solutions. In our work, we proposed the unified Riccati equation expansion method for (3+1)-dimensional Boussinesq model and minimized their dimensional models in shallow water waves to find dark, unique, periodic, and rational solutions. In nonlinear optics, the study of interaction between strong light beams and nonlinear materials is relevant to dark, bright solitons. They are pertinent to phenomena like pulse compression and the creation of supercontinuous. To solve distinct nonlinear partial differential equations that arise in mathematical physics, soliton solutions including dark and brilliant solitons are employed. These solutions shed light on the behaviour of complicated systems. Furthermore, bifurcation analysis exposes every possible nonlinear dynamic system phase portrait. Exact soliton solution structures are shown in Figs. 1, 2, 3, 4 and 5, and phase pictures from bifurcation analysis are displayed in Figs. 6, 7, 8 and 9. Figure 10 represents the chaotic structure with quasi-periodic and sensitivity analysis. The dark soliton solutions are quieter solitary waves than the background whereas the singular soliton solutions are discontinuous derivatives such as peakons and compactions. Periodic solutions repeat throughout time and help with pattern recognition and understanding equation structure. Figures 6, 7, 8 and 10 phase pictures are created by choosing appropriate values from Eq. (37). Solutions formed from equilibrium points and parameter values are shown by variable *A*, where $A>0$ shows the center point, $A<0$ is the saddle point, and $A=0$ is the cuspidal point.

### Graphical description

In this description, we presents the graphical structures in 2-dimensional, 3-dimensional, and density plots.

Figure 1 displays the dark soliton solution for $Q_{1,1}(x,t)$, $Q_{2,1}(x,t)$, and $Q_{3,1}(x,t)$, as described by the their corresponding Eqs. (19), (25), and (31). Figure 2 displays the singular soliton solution for $Q_{1,2}(x,t)$, $Q_{2,2}(x,t)$, and $Q_{3,2}(x,t)$, as described by the their corresponding Eqs. (20), (26), and (32). Figure 3 displays the periodic solution for $Q_{1,3}(x,t)$, $Q_{2,3}(x,t)$, and $Q_{3,3}(x,t)$, as described by the their corresponding Eqs. (21), (27), and (33). Figure 4 displays the periodic solitary wave solution for $Q_{1,4}(x,t)$, $Q_{2,4}(x,t)$, and $Q_{3,4}(x,t)$, as described by the their corresponding Eqs. (22), (28), and (34). Figure 5 displays the rational solution for $Q_{1,5}(x,t)$, $Q_{2,5}(x,t)$, and $Q_{3,5}(x,t)$, as described by the their corresponding Eqs. (23), (29), and (35).

Figure 6 shows the phase portrait for the case-(1) by taking $C>0$ and $D>0$. Figure 7 shows the phase portrait for the case-(2) by taking $C=0$ and $D<0$. Figure 8 shows the phase portrait for the case-(3) by taking $C<0$ and $D<0$. Figure 9 shows the phase portrait for the case-(4) by taking $C>0$ and $D=0$.

## Conclusion

In the present work, we examined the soliton solutions of shallow water waves in a (3+1)-dimensional Boussinesq model. We conducted a qualitative study of the proposed model and derived accurate solutions using the UREE method. We successfully derived dark, solitary, periodic, and rational solutions. We study these solutions with phase depictions to attain a better understanding of the theory of motivation. We utilize bifurcation and chaos theories to comprehend the planar dynamical system and showcase its dependence on physical parameters like quasi-periodic and sensitivity analysis. These innovative results encourage new understandings of wave motion dynamics in mathematical simulations. Using symbolic computing, we solve nonlinear wave problems in multiple fields, such as mathematical physics and engineering. These findings could contribute to understanding how waves propagate in shallow water in oceanography. Future studies on the particular (3+1)-dimensional Boussinesq model may focus on breather wave solutions, hybrid formulations, rogue waves, and multi-lump waveforms.

## Data Availability

All data generated or analyzed during this study are included in this published article.

## References

[1] Mohanty SK, Kravchenko OV, Dev AN (2022). Exact traveling wave solutions of the schamel burgers’ equation by using generalized-improved and generalized $$(g^{\prime }/g)$$ expansion methods. Results Phys..

[2] Kumar S, Rani S (2022). Study of exact analytical solutions and various wave profiles of a new extended (2+ 1)-dimensional Boussinesq equation using symmetry analysis. J. Ocean Eng. Sci..

[3] Khan K, Akbar MA (2023). Study of explicit travelling wave solutions of nonlinear evolution equations. Partial Differ. Equ. Appl. Math..

[4] Fahim MRA, Kundu PR, Islam ME, Akbar MA, Osman M (2022). Wave profile analysis of a couple of (3+ 1)-dimensional nonlinear evolution equations by sine-gordon expansion approach. J. Ocean Eng. Sci..

[5] Roshid M, Bairagi T, Rahman M (2022). Lump, interaction of lump and kink and solitonic solution of nonlinear evolution equation which describe incompressible viscoelastic Kelvin–Voigt fluid. Partial Differ. Equ. Appl. Math..

[6] Jiang Y, Wang F, Salama SA, Botmart T, Khater MM (2022). Computational investigation on a nonlinear dispersion model with the weak non-local nonlinearity in quantum mechanics. Results Phys..

[7] Kharbanda H, Kumar S (2020). Chaos detection and optimal control in a cannibalistic prey-predator system with harvesting. Int. J. Bifurc. Chaos.

[8] Wang S (2023). Novel soliton solutions of cnlses with hirota bilinear method. J. Opt..

[9] Gu Y, Zia SM, Isam M, Manafian J, Hajar A, Abotaleb M (2023). Bilinear method and semi-inverse variational principle approach to the generalized (2+ 1)-dimensional shallow water wave equation. Results Phys..

[10] Raheel M, Zafar A, Cevikel A, Rezazadeh H, Bekir A (2023). Exact wave solutions of truncated m-fractional new Hamiltonian amplitude equation through two analytical techniques. Int. J. Mod. Phys. B.

[11] Onder I, Secer A, Ozisik M, Bayram M (2023). Investigation of optical soliton solutions for the perturbed Gerdjikov–Ivanov equation with full-nonlinearity. Heliyon.

[12] Sivasundari SAS, Jeyabarathi P, Rajendran L (2023). Theoretical analysis of nonlinear equation in reaction-diffusion system: Hyperbolic function method. Eur. J. Math. Stat..

[13] Raza N, Salman F, Butt AR, Gandarias ML (2023). Lie symmetry analysis, soliton solutions and qualitative analysis concerning to the generalized q-deformed sinh-gordon equation. Commun. Nonlinear Sci. Numer. Simul..

[14] Ablowitz MJ, Been JB, Carr LD (2022). Integrable fractional modified Korteweg-devries, sine-gordon, and sinh-gordon equations. J. Phys. A Math. Theor..

[15] Tarla S, Ali KK, Yilmazer R, Osman M (2022). The dynamic behaviors of the Radhakrishnan–Kundu–Lakshmanan equation by Jacobi elliptic function expansion technique. Opt. Quant. Electron..

[16] Khalil TA, Badra N, Ahmed HM, Rabie WB (2022). Bright solitons for twin-core couplers and multiple-core couplers having polynomial law of nonlinearity using Jacobi elliptic function expansion method. Alex. Eng. J..

[17] Islam MT, Akter MA, Gomez-Aguilar J, Akbar MA, Pérez-Careta E (2023). Innovative and diverse soliton solutions of the dual core optical fiber nonlinear models via two competent techniques. J. Nonlinear Opt. Phys. Mater..

[18] Akbulut A, Islam R, Arafat Y, Taşcan F (2023). A novel scheme for smch equation with two different approaches. Comput. Methods Differ. Equ..

[19] Zafar A, Shakeel M, Ali A, Akinyemi L, Rezazadeh H (2022). Optical solitons of nonlinear complex Ginzburg–Landau equation via two modified expansion schemes. Opt. Quant. Electron..

[20] Ali A, Ahmad J, Javed S (2023). Solitary wave solutions for the originating waves that propagate of the fractional Wazwaz–Benjamin–Bona–Mahony system. Alex. Eng. J..

[21] Eldidamony H, Ahmed HM, Zaghrout A, Ali Y, Arnous AH (2022). Mathematical methods for construction new soliton solutions of Radhakrishnan–Kundu Lakshmanan equation. Alex. Eng. J..

[22] Ozisik M (2022). Novel (2+ 1) and (3+ 1) forms of the Biswas–Milovic equation and optical soliton solutions via two efficient techniques. Optik.

[23] Xie J, Wang H, Chen L, Zhao F (2022). Dynamical analysis of fractional oscillator system with cosine excitation utilizing the average method. Math. Methods Appl. Sci..

[24] Alquran M, Alhami R (2022). Convex-periodic, kink-periodic, peakon-soliton and kink bidirectional wave-solutions to new established two-mode generalization of Cahn–Allen equation. Results Phys..

[25] Kumar S, Mann N, Kharbanda H, Inc M (2023). Dynamical behavior of analytical soliton solutions, bifurcation analysis, and quasi-periodic solution to the (2+ 1)-dimensional Konopelchenko–Dubrovsky (kd) system. Anal. Math. Phys..

[26] Kumar S, Mann N (2023). A variety of newly formed soliton solutions and patterns of dynamic waveforms for the generalized complex coupled Schrödinger–Boussinesq equations. Opt. Quant. Electron..

[27] Foroutan M, Manafian J, Ranjbaran A (2018). Lump solution and its interaction to (3+ 1)-d potential-ytsf equation. Nonlinear Dyn..

[28] Kaur L, Wazwaz A-M (2019). Bright-dark lump wave solutions for a new form of the (3+ 1)-dimensional bkp-Boussinesq equation. Rom. Rep. Phys..

[29] Wang H (2018). Lump and interaction solutions to the (2+ 1)-dimensional burgers equation. Appl. Math. Lett..

[30] Liu J-G, Eslami M, Rezazadeh H, Mirzazadeh M (2020). The dynamical behavior of mixed type lump solutions on the (3+ 1)-dimensional generalized Kadomtsev–Petviashvili–Boussinesq equation. Int. J. Nonlinear Sci. Numer. Simul..

[31] Sarwar A, Gang T, Arshad M, Ahmed I, Ahmad M (2023). Abundant solitary wave solutions for space-time fractional unstable nonlinear Schrödinger equations and their applications. Ain Shams Eng. J..

[32] Bilal M, Shafqat-Ur-Rehman JA (2022). Analysis in fiber bragg gratings with kerr law nonlinearity for diverse optical soliton solutions by reliable analytical techniques. Modern Phys. Lett. B.

[33] Wazwaz A-M (2013). Multiple soliton solutions for an integrable couplings of the Boussinesq equation. Ocean Eng..

[34] Hossain MD, Alam MK, Akbar MA (2018). Abundant wave solutions of the Boussinesq equation and the (2+ 1)-dimensional extended shallow water wave equation. Ocean Eng..

[35] Ozisik M, Secer A, Bayram M (2023). Soliton waves with the (3+ 1)-dimensional Kadomtsev–Petviashvili–Boussinesq equation in water wave dynamics. Symmetry.

[36] Chaichitehrani N, Li C, Xu K, Hestir EL, Allahdadi MN (2023). Sediment dynamics over a dredge pit during summer fair weather conditions: A numerical study for sandy point, west flank of the Mississippi river. Ocean Eng..

[37] Ozdemir N (2022). Optical solitons for Radhakrishnan–Kundu–Lakshmanan equation in the presence of perturbation term and having kerr law. Optik.

[38] Yao S-W, Nuruzzaman M, Kumar D, Tamanna N, Inc M (2023). Lump solutions to an integrable (3+ 1)-dimensional Boussinesq equation and its dimensionally reduced equations in shallow water. Results Phys..

[39] Wazwaz A-M, Kaur L (2019). New integrable Boussinesq equations of distinct dimensions with diverse variety of soliton solutions. Nonlinear Dyn..

[40] Yao S-W, Tariq KU, Inc M, Tufail RN (2023). Modulation instability analysis and soliton solutions of the modified bbm model arising in dispersive medium. Results Phys..

[41] Yang X, Fan R, Li B (2020). Soliton molecules and some novel interaction solutions to the (2+ 1)-dimensional b-type Kadomtsev–Petviashvili equation. Phys. Scr..

[42] Huang L, Yue Y, Chen Y (2018). Localized waves and interaction solutions to a (3+ 1)-dimensional generalized kp equation. Comput. Math. Appl..

